# Air pollution and public health in Latin America and the Caribbean (LAC): a systematic review with meta-analysis

**DOI:** 10.1186/s43088-022-00305-0

**Published:** 2022-09-30

**Authors:** Danladi Chiroma Husaini, Kyle Reneau, Daren Balam

**Affiliations:** Pharmacy Program, Department of Allied Health, Faculty of Health Sciences, University of Belize, Belmopan Central Campus, Belmopan, Belize

**Keywords:** Air pollution, Particulate matter, Environmental pollution, Air quality, Public health, Latin America and the Caribbean

## Abstract

**Background:**

Over the years, air pollution has garnered increased attention from researchers who continue to provide studies and suggestive data that prove there is an ever-increasing risk of air pollution on the health of humans, terrestrial, and aquatic animals. A measurement involved in the quantity of certain traceable particles within the air, namely: Particulate Matter (PM) 2.5 and 10, ozone (O_3_), Nitrogen dioxide (NO_2_), sulfur dioxide (SO_2_), and carbon monoxide (CO) emissions, all converted to Air Quality Index. Most studies are predominantly from developed nations with limited research conducted in developing nations such as those in Latin America and the Caribbean.

**Main body:**

In this systematic review, we examined the impact of air pollution on public health. A database search produced 1,118 studies, of which four were selected for a quantitative meta-analysis that explored hazard ratios concerning exposure to elevated levels of PM2.5. The meta-analysis results show that exposure to PM2.5 increases the risk of an adverse health event by as much as 2% five days after exposure. Results also indicated a consensus on the negative impacts of air pollution on public health. The results also suggest that more can be done within the region to combat or at the very least minimize the impact of air pollution to public health.

**Conclusion:**

The pooled data from the studies reviewed show that there is an increased risk of an adverse health event on the day of exposure to PM2.5 and every subsequent day after exposure. A pattern exists between hospitalization and air pollution due to increased susceptibility to respiratory infections and asthma development. Combating the harmful effects of air pollution should be a top priority in Latin America and the Caribbean.

## Background

The contamination of water, air, and land through the introduction of harmful biological, chemical, and physical agents is termed pollution. Pollution negatively impacts the environment and public health, leading to morbidity and mortality [1]. Lack of awareness of the different forms of pollution, poor policies and legislation, inadequate or lack of enforcement, and poverty generally make the pollution burden greater in developing countries than in developed countries [2]. Although the negative impact of water and land pollution on public health cannot be underrated, the environmental and public health impacts of air pollution are dire, especially in developing countries [3]. Air pollution is defined as an atmospheric condition in which substances are present in the air above normal levels, creating measurable adverse effects, particularly on animals, humans, and vegetation [4]. Air pollution poses a significant risk to the health of the world’s population, with even short-term exposure linked to numerous respiratory diseases and increased hospitalization rates [5]. Additionally, air pollution affects pollution levels in water and soil via precipitation, potentially leading to harmful chemicals present in produce and livestock used for human consumption [6]. In a recent review, Manisalidis et al. [5] highlighted the numerous health effects of air pollution, including respiratory, reproductive, cardiovascular, neurological, and specific cancers. In addition, environmental impacts were reported to include climate change, ozone depletion, haze, acid rain, and heat-related public health issues. Through the depletion of the ozone layer and global warming, the quality of life on Earth is adversely affected by air pollution. Gaseous pollutants such as carbon monoxide (CO), volatile organic compounds (VOCs), nitrogen oxides, and sulfur oxides enter the environment through various means and distributes within the atmosphere. These primary pollutants are released into the environment primarily due to mining, exploration, population growth, urbanization, industrialization, and natural disasters [2]. In addition, solid waste disposal contributes more than 4.2% of the total air pollution, as reported in EPA emission totals [7, 8].

Air pollution is generally measured using particulate matter (PM2.5 and PM10), ozone (O_3_), nitrogen dioxide (NO_2_), sulfur dioxide (SO_2_), and carbon monoxide (CO) emissions and converted to an Air Quality Index (AQI). An AQI of 100 or below is acceptable, with values above 100 ranging from unhealthy for sensitive populations to hazardous. Air pollutants can come from numerous sources, including traffic, industry, and energy production, which places a higher risk of exposure to air pollution in urban areas [9]. Lower- and middle-income countries, which most countries in Latin America and the Caribbean fall into, are experiencing a boom in urbanization, putting additional risk on a population with an already struggling healthcare system. The World Health Organization (WHO) [10] reported that air pollution is associated with increased mortality and morbidity, killing an estimated seven million people worldwide; its increase does not bode well for public health. Within the last decade, numerous research has found substantial data showing air pollution is associated with a broad number of disease outcomes, ultimately leading to premature death from ischemic heart disease, chronic obstructive pulmonary disease (COPD), lung cancer, and acute lower respiratory infections [11]. Recent reviews of COVID-19 data indicates that air pollution may contribute significantly to higher rates of COVID-19 infections and mortality and its transmission [3, 12]. Relatedly, correlations between air pollution, COVID-19, and rheumatic diseases were established and reported [13–15]. Cardiovascular and lung diseases and other serious health challenges have also been reported due to air pollutants. People with pre-existing noncommunicable diseases, older adults, pregnant women, children, and persons living in low socioeconomic communities are more vulnerable to air pollution and its attendant negative health impacts [16].

Although many developed and industrialized nations have researched and reported on the impacts of air pollution on public health, within Latin America and the Caribbean, this area has been somewhat underexplored despite the region having over 100 million residents living in areas exposed to air pollution levels exceeding the World Health Organization guidelines [17]. Air pollution places a significant risk to the public's health, from early disease development to premature death, which also increases health-related expenditure and resources. Thus, it is essential for Latin America and the Caribbean to have more and continuing research in this area to influence policy, reduce public health risk, and reallocate resources and expenses to more needed areas. Although studies show links between air pollution and disease in Latin America and the Caribbean, a systematic review of the evidence of air pollution and its effects on public health is lacking.

Using a systematic review methodology, this paper reviewed the impact of air pollution on public health within Latin America and the Caribbean.

## Main text

### Methodology

Relevant studies were identified through electronic search of Google Scholar, EBSCOHOST, HINARI, Scielo, PubMed, and Scopus databases. Search combined terms included 'air pollution,' 'public health,' 'Latin America,' 'Caribbean,' 'Central America,' and 'South America.' Rayyan systematic review software was used for uploading and sorting study references.

### Risk of bias in individual studies

**Fig. 1:**
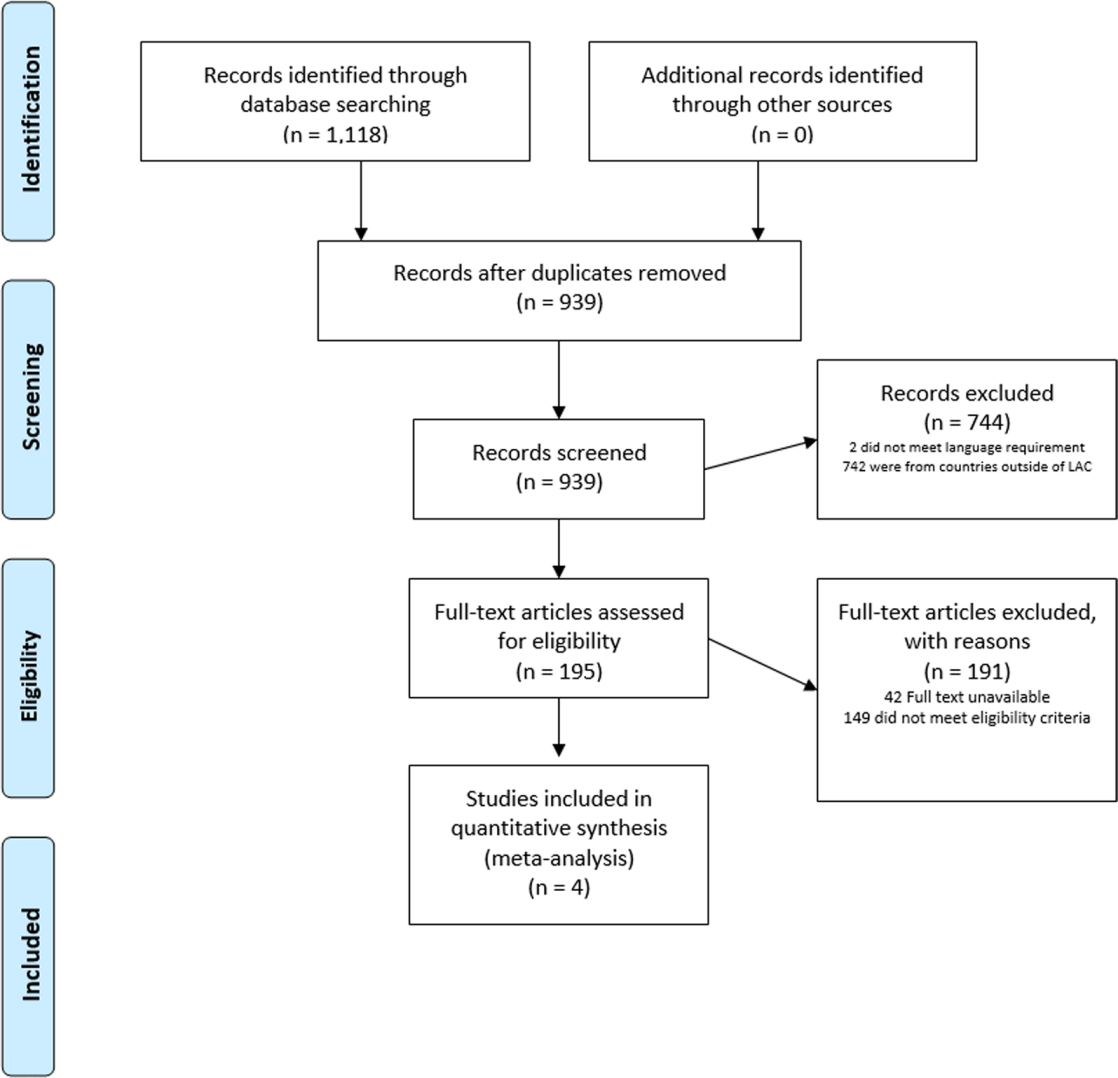
Study identification and selection process

Two independent reviewers conducted a qualitative assessment on which studies to include and exclude and classify studies as low or high risk of bias to reduce bias (Table 1, Fig. 1). Additionally, publication bias for included studies was assessed using a funnel plot.

**Table 1:** Criteria for inclusion and exclusion

Inclusion	Exclusion
Peer-reviewed English Language	Non-English
Empirical studies	Literature reviews
Published < 10 years	Published > 10 years
Focus on LAC countries	Focuses on countries outside of LAC
Quantitative data	Qualitative data
Casual Research	Laboratory Research
Reported quantitative measurements of minimum 5-day lag	Data not presented in a 5-day lag structure

### Summary measures

**Fig. 2:**
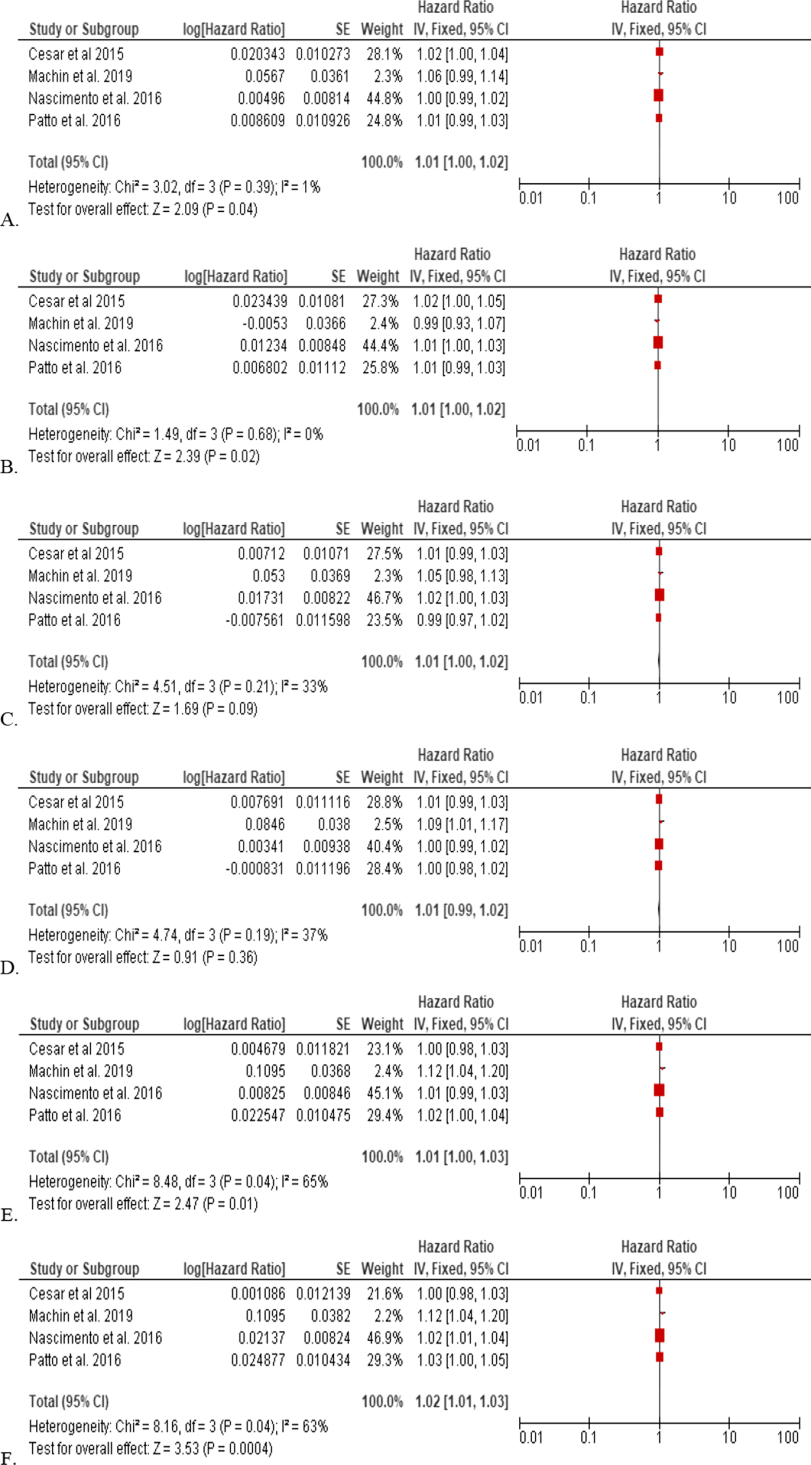
Forest plots of hazard ratios **A** Lag0, **B** Lag1, **C** Lag2, **D** Lag3, **E** Lag4, **F** Lag5

The statistical analysis software, Review Manager 5.4.1, was used to calculate the summary effects of the lag structure (Fig. 2). For analysis, all corresponding lags were grouped for analysis of exposure to PM2.5. Cochrane Q test (significance level: 0.1) and *I*^2^ statistic were used to test statistical heterogeneity. *I*^2^ statistic quantifies heterogeneity by calculating the proportion of variation that occurred by heterogeneity rather than by chance [18]. For *I*^2^, values ranging from 0 to 30, 30 to 50, and > 50 indicated low, moderate, and high heterogeneity, respectively.

## Results

Database searches produced 1118 studies, of which four were selected for quantitative meta-analysis (Fig. 1). All four studies originated from Brazil and explored exposure to air pollution and its corresponding health risks on the day of exposure and up to at least five days after. Air pollution assessments include CO, O_3_, NO_X,_ and PM2.5_,_ but only PM2.5 data was used for statistical analysis as it was the only assessment data common among the four studies (Table 2, Fig. 2).

**Table 2:** Characteristics of included studies

References	Country	Country income	Research aim	Exposure assessment	Outcome
[19]	Brazil	Upper middle income	The objective of this systematic review was to evaluate the effect of exposure to particulate matter on hospitalizations in relation to certain respiratory diseases amongst residents in Volta Redonda (RJ)	PM2.5	There were 752 hospitalizations in 2012 and the average concentration of PM2.5 was 17.2 μg/m^3^; the effects of exposure were seen to be significant at lag 2 (RR = 1.017), lag 5 (RR = 1.022) and lag 7 (RR = 1020). A decrease in PM2.5 concentration of 5 μg/m3 could lower admissions by 76 cases and decrease spending by up to R$84,000 annually
[20]	Brazil	Upper middle income	This study aimed to estimate the association between hospitalizations due to asthma and air pollutants	CO, O_3_, NO_X_, PM2.5	Exposure to NO_x_ was associated with mortality owing to respiratory diseases from 2011 to 2012: relative risk (RR) = 1.035 (95% confidence interval [CI] 1.008–1.063) for lag 2, RR = 1.064 (95%CI 1.017–1.112) lag 3, RR = 1.055 (95%CI 1.025–1.085) lag 4, and RR = 1.042 (95%CI 1.010–1.076) lag 5. A 3 mg/m3 reduction in NO_x_ concentration decreased 10–18% points in the risk of death caused by respiratory diseases. Even at NO_x_ concentrations below hazardous standards, there is an association with deaths and respiratory diseases
[21]	Brazil	Upper middle income	The aim was to determine the effects of exposure to fine particulate matter in elderly hospitalizations owing to respiratory diseases in the South of the Brazilian Amazon	PM2.5	Significant associations between exposure to PM_2.5_ and hospitalizations in lags 3 and 4 in 2012 were observed. About thirty-two percent hospitalization risk increase, with an increase of 3.5 mg/m3 of PM2.5 concentrations leading to an increase of 188 in the total number of hospitalizations, creating an expense of more than US$ 96,000
[22]	Brazil	Upper middle income	This systematic review estimated the role of exposure to fine particulate matter in hospitalizations due to pneumonia and how a reduction may affect the number of these hospitalizations and costs	PM2.5	Exposure to air pollutants was associated with hospitalization four and five days after exposure and increased hospitalization cost from 2011 to 2013

Meta-analysis of the hazard ratios from the day of exposure to PM2.5 shows a 1% increase in the risk of an adverse health event for the day of exposure up to 4 days after exposure, with day 5 showing a 2% increase.

## Discussion

We conducted a systematic review to explore the impact of air pollution on public health in Latin America and the Caribbean. The aim was to determine if the grouped data expressed the same results and were, for the most part, in consensus with each other. There is an understanding that air pollution negatively impacts health, but does the available data support this assumption, particularly within the Latin American and Caribbean regions?

Using hazard ratios after exposure to PM2.5, the pooled data suggests an increased risk of an adverse health event after exposure to air pollution (Table 2). These findings resonate with studies in developed countries [23]. PM2.5 is fine particulate matter in the air that is less than 2.5 µm in diameter, with the concentrations of PM2.5 being used in calculating the Air Quality Index. PM2.5 has been linked to numerous respiratory issues, as it can penetrate deeply into the lungs and significantly impair lung function [24]. The European Environment Agency (EEA) [25] reported that particulate matter ranks among the top 5 most harmful air pollutants. The pooled data from the four studies showed an increased risk of an adverse health event on the day of exposure to PM2.5 and every subsequent day after exposure (Table 2, Fig. 2). Of note was that the increased risk advanced to 2% on the fifth day after exposure, but with a moderate heterogeneity percentage, suggesting some level of 'uncombinablity' (Table 2, Fig. 2). Despite this, the evidence is still strong in response to the risk associated with exposure to air pollution, showing that the risk exists not only on the day of exposure but can adversely affect public health in the near future.

Furthermore, a pattern was established between hospitalization and air pollution in the four studies evaluated. Using an ecological time-series study, Cesar et al. [20] conducted a study to estimate the association between hospitalization caused by respiratory diseases and air pollutants. By comparing deaths from respiratory diseases and estimated daily levels of air pollutants over an approximate one-year period, their study established an association between exposure to Nitrogen oxides (NO_x_) and deaths from respiratory diseases. Similarly, the United States Environmental Protection Agency (US-EPA) [16] documented that exposure to NO_x_, such as NO_2_, even over short periods, can aggravate respiratory diseases, such as asthma, which leads to increased respiratory symptoms, hospital admission, and emergency room visits. Furthermore, the US-EPA also reported that more prolonged exposure to elevated concentrations of NO_2_ might contribute to a potential increase in susceptibility to respiratory infections and the development of asthma.

In another ecological time-series study, Nascimento et al. [19] reported that respiratory diseases are influenced by exposure to numerous air pollutants, such as NO_2_, particulate matter less than PM10, carbon monoxide, and sulfur dioxide, leading to hospitalizations. The study was conducted over one year, with data showing that an increase in PM2.5 concentrations significantly increased the risk of hospitalization due to respiratory diseases such as pneumonia, acute bronchitis, bronchiolitis, and asthma.

Evidence suggests that those with existing respiratory disease conditions are at an increased risk of adverse health effects due to exposure to air pollutants. Machin et al. [21] identified and reported the effects of exposure to fine particulate matter and CO on the number of hospitalizations due to respiratory diseases in the elderly (60 years and older). Their report demonstrated a significant association between exposure to these particles and hospitalizations, with an increased risk of hospitalization of 31.8%. An increased concentration of PM2.5 demonstrated a significant negative impact on respiratory disease exacerbation.

The review results resonate with other studies done worldwide. Slama et al. [26] reported a positive association between ambient air pollution and hospitalization, where PM2.5 and PM10 had the most significant effect. All four studies looked primarily at hospitalization associated with visits for respiratory ailments; however, exposure to particulate matter has other detrimental effects on health. Zhang et al. [27] reported that particulate matter exposure increased hospitalization visits for arrhythmia, high blood pressure, cerebrovascular disease, and ischemic heart disease. This implication to other health-related hospitalizations shows that many individuals with pre-existing health conditions are susceptible to short-term air pollution exposure, increasing public health risk, and public health expenditure, both in the private and public sectors.

The results of this systematic review can help guide policy changes regarding the rise in urbanization within the Caribbean and Latin American regions. However, the review results can also benefit healthcare providers who can educate patients on the health risk of air pollution and implement strategies at the public health level to deal with increases in adverse health events to Unhealthy Air Quality Index levels. Combating the harmful effects of air pollution should be a priority within the LAC region, as it can potentially reduce public health costs by millions in each respective country. An essential first step is to examine available data, which this systematic review provides. Additionally, more studies within the region ought to be done so that additional data can be used to design tailored policies for each country, as what may work for Jamaica may not work for Ecuador.

## Limitations

Some limitations of this study include a bias risk, as all study data that met the inclusion criteria originated in Brazil. This limitation is partly a result of few regional studies on air pollution and public health, primarily due to the inaccessibility of daily Air Quality Index data. Another limitation is that only PM2.5 was accessed, not the other measurements included in the Air Quality Index. A multivariate analysis would have possibly yielded more robust results, but the researchers were unable to account for the 'uncombinability’ of such a data set. Finally, the various assessment data in each study could not be grouped as one, as the software used could not account for the multivariate nature of such a data set.

## Conclusion

With a fast rate of development within Latin America and the Caribbean, it is crucial to outline and provide continuous research on the consequences and impact of air pollution on the population of these developing nations. Though there is an overwhelming lack of data and studies on air pollution within the region, the available data revealed a negative impact of air pollution on public health from the day of exposure up to five days after exposure. The 1% increase in the exposure rate to 2% by day 5 is evident in the results of the hazard ratios. Countries within Latin America and the Caribbean need to examine their developmental impacts on air pollution, how it affects public health, and how it can be effectively managed.

## Data Availability

All data generated or analyzed during this study are included in this published article.

## Abbreviations

AQI: Air quality index
CO: Carbon monoxide
COPD: Chronic obstructive pulmonary disease
EEA: European Environment Agency
LAC: Latin America and the Caribbean
NO_2_: Nitrogen dioxide
NO_x_: Nitrogen oxides
O_3_: Ozone
PM: Particulate matter
SO_4_: Sulfur dioxide
USEPA: United States Environmental Protection Agency
WHO: World Health Organization
